# Alleviated Oxidative Damage by *Taraxacum officinale* through the Induction of Nrf2-MAPK/PI3K Mediated HO-1 Activation in Murine Macrophages RAW 264.7 Cell Line

**DOI:** 10.3390/biom9070288

**Published:** 2019-07-18

**Authors:** Hyun-Seo Yoon, Chung Mu Park

**Affiliations:** 1 Department of Dental Hygiene, Dong-Eui University, Busan 47340, Korea; 2 Department of Clinical Laboratory Science, Dong-Eui University, Busan 47340, Korea

**Keywords:** *Taraxacum officinale*, Heme Oxygenase-1, NF-E2-Related Factor 2, Mitogen-Activated Protein Kinases

## Abstract

*Taraxacum officinale* has been consumed as a folk remedy due to its diverse physiological activities. This study aimed to investigate the antioxidative potential of *T. officinale* water extract (TOWE) and ethanol extract (TOEE) against oxidative stress and compare their molecular mechanism via the induction of heme oxygenase-1 (HO-1) in RAW 264.7 cells. The antioxidative activity was evaluated through the radical scavenging assay, the cytoprotection assay against oxidative damage, and Western blot analysis. Both extracts dose-dependently induced HO-1 expression without any cytotoxicity in accordance with the activation of a transcription factor, nuclear factor-erythroid 2 p45-related factor 2 (Nrf2). In addition, TOWE induced HO-1 expression through the phosphorylation of phosphoinositide 3-kinase (PI3K)/Akt and c-Jun NH_2_-terminal kinase (JNK), while TOEE activated HO-1 by PI3K/Akt phosphorylation. In order to identify the antioxidative potential by HO-1 induction, oxidative damage-caused cell death by tert-butyl hydroperoxide (t-BHP) was significantly attenuated by both extracts. Their antioxidative potential was confirmed by HO-1 selective inducer and inhibitor, cobalt protoporphyrin (CoPP), and tin protoporphyrin (SnPP), respectively. These results indicate that TOWE and TOEE potently alleviated oxidative damage via the induction of Nrf2/MAPK/PI3K mediated HO-1 induction in RAW 264.7 cells.

## 1. Introduction

Excess generation of intracellular reactive oxygen species (ROS) in response to xenobiotics, drugs, cytokines, and environmental stress can cause damage through the modification or degradation of cellular components, including DNA, protein, lipids, and carbohydrates, which contributes to the tissue injury in the context of a variety of disease state [1]. Oxidative stress is highly related to the progress of diverse diseases, such as age-related macular degeneration, Parkinson’s disease, alcoholic hepatitis, and cardiovascular diseases [2]. In order to eliminate the oxidative stress, cells have developed their own defensive mechanisms through the induction of antioxidative enzymes, such as superoxide dismutase (SOD), heme oxygenase-1 (HO-1), and NAD(P)H: quinone oxidoreductase (NQO1). HO-1 is one of the metabolizing enzymes and converts heme to biliverdin, carbon monoxide, and free iron. These catabolites have exhibited potent protective activities against oxidative stress and inflammation [3]. HO-1 is regulated by a nuclear factor-erythroid 2 p45-related factor 2 (Nrf2), which can be activated in response to oxidative stress [4]. Activated Nrf2 can translocate into the nucleus and bind the antioxidant response element (ARE), which can lead the gene expression of various antioxidative enzymes including HO-1 [5]. Additionally, numerous lines of research indicate that multiple signaling molecules such as mitogen-activated protein kinase (MAPK) and phosphatidylinositol 3-kinase (PI3K) play a critical role in the regulation of the Nrf2 activation [6,7,8]. Therefore, Nrf2 plays an important role in transcriptional regulation of antioxidative genes and Nrf2 mediated signaling pathway can be mediated by MAPKs and PI3K [9].

Various phytochemicals have proposed as potential nutraceuticals to attenuate the progress of the oxidative stress-induced disorders [10]. Thus, the ingestion of food containing abundant antioxidants is considered to be an important way to counterpart oxidative stress. Among plant resources, *Taraxacum officinale* has consumed as a functional food or herbal therapeutic agent due to its potentials for choleretic, diuretic, antioxidative, and anti-inflammatory activities [11]. Especially, antioxidative activity of *T. officinale* extracts from various parts of the plant was analyzed through numerous cell lines and animal models. Flower extract inhibited peroxyl radical-induced intracellular oxidation in RAW 264.7 cells and flavonoids from *T. officinale* protected V79-4 hamster lung fibroblast cells from free radical-induced cytotoxicity [12,13]. Polysaccharides from *T. officinale* (TOP 1 and 2) exhibited potent antioxidative potential against tert-butyl hydroperoxide (t-BHP) induced oxidative damage through the induction of Nrf2-mediated HO-1 expression in RAW 264.7 cells [14]. In addition, root and leaf extracts of *T. officinale* administration improved activities of various antioxidative enzymes in hypercholesterolemia-induced rabbit [15]. *T. officinale* water extract protected hepatic injury through the induction of antioxidative enzymes activities in carbon tetrachloride (CCl_4_)-intoxicated SD rat [16]. A leafy vegetable mixture containing the *T. officinale* as one kind of ingredients also increased antioxidative enzymes activities in high fat and high cholesterol administered mice [17]. Although there are a lot of studies on *T. officinale*, the study on the induction of HO-1 enzyme has not been conducted yet. The present study aimed to analyze the antioxidative potential of *T. officinale* extracts through the induction of HO-1, one of its metabolizing enzymes, and its mechanism in RAW 264.7 cells.

## 2. Materials and Methods 

### 2.1. Reagents

Sodium dodecyl sulfate (SDS) and dimethyl sulfoxide (DMSO) were obtained from the Sigma-Aldrich (St. Louis, MO, USA). Cobalt protoporphyrin (CoPP) and tin protoporphyrin (SnPP) were purchased from Frontier Scientific (Logan, UT, USA). Antibodies against HO-1, Nrf2, poly (ADP-ribose) polymerase (PARP), phospho-extracellular signal-regulated kinase (ERK), ERK, phospho-c-Jun NH_2_-terminal kinase (JNK), JNK, phospho-p38, p38, phospho-Akt, Akt, actin, and horseradish peroxidase (HRP)-conjugated anti-rabbit IgG were offered from Cell Signaling Technology (Danvers, MA, USA).

### 2.2. Preparation of T. officinale Extracts

*T. officinale* was obtained from Mindleh Food (Uiryeong, Korea). Washed and dried *T. officinale* leaves were ground to a fine mesh powder. *T. officinale* leaves water extract (TOWE) and *T. officinale* ethanol extract (TOEE) were prepared as follows. One hundred grams of *T. officinale* powder was extracted with 1 L of water at 100 °C and ethanol at 80 °C for 4 h in a double boiler. After extraction, both extracts were filtered (Whatman paper No. 4). Then, TOWE was concentrated by lyophilization (Biotron, Bucheon, Korea). Lyophilization is the water removal process that usually consists of three steps: the first stage is the freezing phase that freezes the product, the second stage is the sublimation phase where the lowered pressure and the heat are added to sublimate water, and the adsorption phase is the removal step of the ionically-bound water by breaking bonds between the material and the water molecules. Most materials can be dried to 1–5% residual moisture when the freeze-drying process is completed. In addition, TOEE was prepared by rotary evaporation (Buchi, St. Gallen, Switzerland). The recovery rates were 22.5% and 16.3%, respectively.

### 2.3. Cell Culture and Treatment

The RAW 264.7 cell line was supplied from ATCC (TIB-71, Manassas, VA, USA) and maintained in Dulbecco’s modified eagle medium (DMEM) supplemented with 10% fetal bovine serum (FBS), 2 mM L-glutamine, 100 U/mL of penicillin, and streptomycin at 37 °C in a humidified chamber containing 5% CO_2_.

### 2.4. Cell Viability

Cytotoxicity was determined using the WST-1 cell proliferation assay kit purchased from Daeil Laboratory Service (Seoul, Korea). RAW 264.7 cells were seeded in 24-well plate and attached for 24 h. Then the cells were treated with agents and selective inhibitors at indicated concentrations. After treatment, the cells were incubated with WST for 1 h at 37 °C. The absorbance of generated formazan was measured at 450 nm with a multiplate reader (Bio-Rad, Hercules, CA, USA). 

### 2.5. Intracellular ROS Formation Assay

In order to measure the intracellular ROS concentration, a dichloro-dihydro-fluorescein diacetate (DCFH-DA) assay was applied [18]. RAW 264.7 cells were stained with 50 μM of DCFH-DA for 2 h and pre-incubated with TOWE and TOEE for 2 h. Then, cells were treated with 1 μg/mL of lipopolysaccharide (LPS) for 18 h. Fluorescence was measured at 485 nm excitation and 530 nm emission wavelength (Bio-Tek Instruments Inc., Winooski, VT, USA), respectively.

### 2.6. Isolation of Nuclear Protein

The nuclear protein was extracted by the NE-PER Nuclear and Cytoplasmic Extraction Reagents kit (Thermo Fisher Scientific, Waltham, MA, USA). RAW 264.7 cells (5 × 10^6^ cells/dish) were seeded in 100-mm dishes and incubated with indicated concentrations of TOWE or TOEE. Then, cells were harvested by centrifuging at 500× *g* for 5 min. Then, ice-cold cytoplasmic extraction reagent (CER) I was added to the cell pellet and vortexed vigorously for 15 sec to resuspend the cell pellet. Cells were incubated on ice for 10 min. Ice-cold CER II was added to the tube and vortexed for 5 sec. Cells were incubated for 1 min and centrifuged at 13,000× *g*. The supernatant was then transferred to a new tube. 

### 2.7. Western Blot Analysis

RAW 264.7 cells (5 × 10^6^ cells/100 mm dish) were incubated with various concentrations of TOWE or TOEE for the indicated durations of time. The cells were harvested into 0.5 mL of ice-cold protein extraction solution (M-PER, Thermo Fisher Scientific) and lysed for 10 min on the ice. Then, the lysis buffer was centrifuged at 13,000× *g* for 5 min. The supernatant was transferred to a new tube and applied to western blot analysis. The protein samples (50 μg) were separated on a 10% SDS-polyacrylamide gel and electrotransferred to polyvinylidene fluoride (PVDF) membrane (Bio-Rad). The membranes were blocked for 1 h at room temperature with 5% non-fat dry milk in Tris-buffered saline with Tween20 (TBST) solution. Then, the membrane was incubated with a primary antibody with gentle agitation overnight at 4 °C. After the overnight hybridization, the membranes were washed with TBST and then incubated with horseradish peroxidase (HRP) conjugated anti-rabbit IgG for 2 h at room temperature. The membranes were developed with an ECL substrate solution (Santa Cruz Biotechnology, Dallas, TX, USA), and the data were quantified using the Gel Doc EQ System (Bio-Rad).

### 2.8. Statistical Analysis

Oneway ANOVA with Duncan’s multiple range test was conducted by the SPSS program (version 25.0, SPSS Inc., Chicago, IL, USA). All data were expressed as mean ± the standard deviation of three independent experiments. *P* < 0.05 was considered as statistically significant.

## 3. Results and Discussion

### 3.1. TOWE and TOEE Exhibited Potent Antioxidative Activity in LPS-Stimulated RAW 264.7 Cells

**Figure 1:**
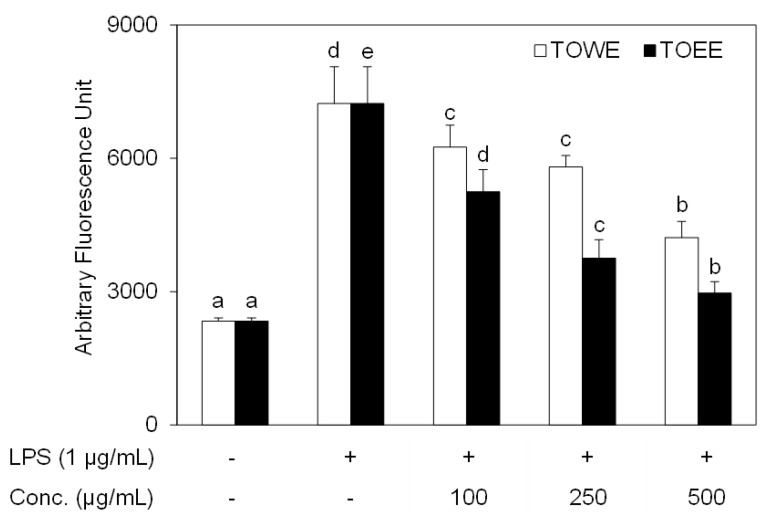
TOWE and TOEE scavenged LPS-induced ROS formation in RAW 264.7 cells. The cells were incubated with 50 μM of DCFH-DA for 2 h. Then, either TOWE or TOEE was treated for 2 h and LPS was served (1 μg/mL) for 30 min to generate ROS. The data represent the mean ± SD of triplicate experiments. The values sharing the same superscript are not significantly different at *p* < 0.05 by Duncan’s multiple range test. TOWE, T. *officinale* water extract; TOEE, T. *officinale* ethanol extract; LPS, lipopolysaccharide; conc, concentration.

This study aimed to investigate the antioxidative potential of *T. officinale* extracts through HO-1 induction and compare antioxidative mechanisms in RAW 264.7 cells. First of all, a radical scavenging assay was conducted in order to analyze whether both extracts have antioxidative activity. Figure 1 shows that TOWE and TOEE treatments dose-dependently inhibited LPS-induced ROS production without any cytotoxicity (data not shown), which suggests that both extracts might be promising candidates as antioxidants. In this study, one of the antioxidative enzymes, HO-1, was analyzed in order to determine whether both TOWE and TOEE have the induction potential of HO-1 enzyme in RAW 264.7 cells.

### 3.2. TOWE and TOEE Induced HO-1 Expression in Accordance with the Activation of Nrf2 in RAW 264.7 Cells

**Figure 2:**
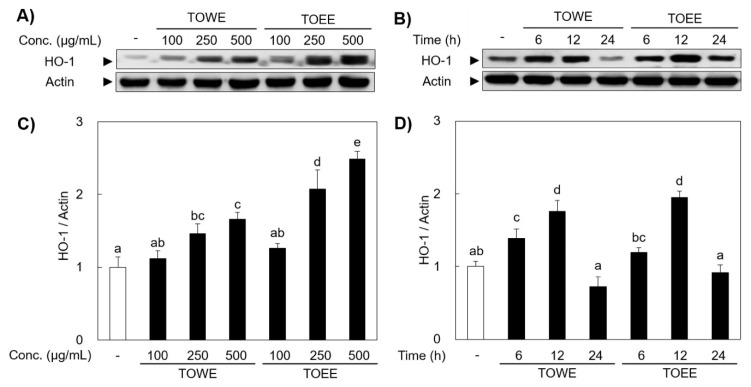
TOWE and TOEE induced HO-1 expression in RAW 264.7 cells. (**A**) RAW 264.7 cells were incubated with diverse concentrations of TOWE and TOEE for 12 h to find out the optimal dose for HO-1 induction. (**B**) The cells were treated with 500 μg/mL for indicated various durations (0, 6, 12, and 24 h). HO-1 expression was potently induced at 500 μg/mL for 12h treatment in RAW 264.7 cells, which was analyzed by Western blot analysis. (**C**,**D**) The relative induction ratio of the HO-1 protein expression was quantified by densitometry and actin was used as an internal control. The data represent the mean ± SD of triplicate experiments. The values sharing the same superscript are not significantly different at *p* < 0.05 by Duncan’s multiple range test. HO-1, heme oxygenase-1.

Diverse concentrations and treatment time were tested to find the optimal condition for HO-1 induction in RAW 264.7 cells. As shown in Figure 2A, TOWE and TOEE dose-dependently induced HO-1 expression. In addition, the optimal time for HO-1 induction was 12 h at 500 μg/mL of TOWE and TOEE treatment (Figure 2B). To sum up, a 500 μg/mL dose of TOWE and TOEE treatment was the most potently induced HO-1 expression at 12 h of treatment time. In a comparison of both extracts, TOEE potently scavenged ROS and elevated HO-1 induction, moreso than those of TOWE. Many researchers have tried to identify bioactive compounds of *T. officinale*, which were evaluated as luteolin, chicoric acid, chlorogenic acid, and chrysoeriol [19,20]. Previous studies showed that extracts from organic solvents including ethanol and methanol usually high antioxidative activity than water extract [21,22]. Concentrations of total phenol and functional compounds such as luteolin and chicoric acid were much more contained in *T. officinale* ethanol and methanol extracts (TOEE and TOME) than those of TOWE [23]. These bioactive compounds have reported they exert potent anti-inflammatory and antioxidative activities [23]. Therefore, TOEE exhibited more strong antioxidative activity than that of TOWE in RAW 264.7 cells.

Induced HO-1 degrades from heme to its by-products such as carbon monoxide, free iron, and biliverdin, which have exhibited to provide a protective function against the inflammatory stimuli and oxidative stress [24]. Carbon monoxide, a gaseous metabolite of heme catabolism, exhibits regulatory effects of vasodilation and proinflammatory responses [25]. Iron is an essential nutrient metal ion which plays a role in mammalian cell growth and proliferation. Ferritin acts as an intracellular iron repository and largely generated ferritin inhibited the ROS accumulation in response to oxidative stress [26]. Biliverdin conversion of bilirubin also exhibited antioxidative activity through inhibited lipid peroxidation and scavenged peroxy radicals [27].

**Figure 3:**
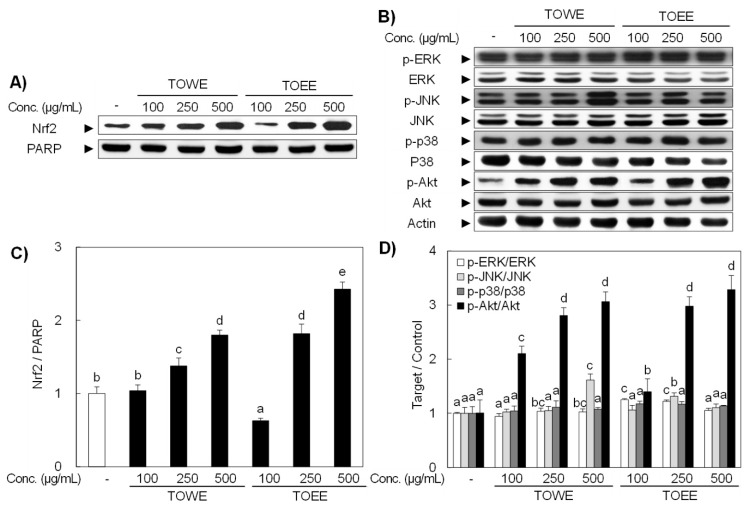
TOWE and TOEE induced nuclear translocation of Nrf2 as well as HO-1 expression through the regulation of JNK and PI3K/Akt signaling pathways in RAW 264.7 cells. (**A**) RAW 264.7 cells were treated with various concentrations of TOWE and TOEE for 12 h. Then, the translocated status of Nrf2 was determined from the nuclear extract by Western blot analysis. (**B**) The cells were treated with indicated doses for 4 h to activate signaling molecules. TOWE triggered the phosphorylation of JNK and Akt, while TOEE only phosphorylated Akt in RAW 264.7 cells. The phosphorylated status of MAPKs and Akt were also analyzed by Western blot analysis. (**C**,**D**) The relative induction ratio of the Nrf2 and HO-1 expression was quantified by densitometry as well as PARP and actin were used as internal controls. The data represent the mean ± SD of triplicate experiments. The values sharing the same superscript are not significantly different at *p* < 0.05 by Duncan’s multiple range test. Nrf2, nuclear factor-erythroid 2 p45-related factor 2; PARP, poly (ADP-ribose) polymerase; ERK, extracellular signal-regulated kinase; JNK, c-Jun NH_2_-terminal kinase.

Nrf2 is sequestered in the cytoplasm as an inactive form anchored by a repressor, Kelch-like ECH-associated protein 1 (Keap1) that is a cytoskeleton binding protein. Extracellular stimuli including oxidative stress and antioxidants provide signals to make Nrf2 free from inhibitory protein. Dissociated Nrf2 from its repressor, once translocates into the nucleus, binds to the ARE or electrophile/stress response elements (EpRE/StRE) located in the promoter region of genes encoding various antioxidative enzymes including HO-1 [28]. To investigate whether TOEE and TOWE can induce Nrf2 activation in RAW 264.7 cells, Western blot analysis was applied and Nrf2 expression was dose-dependently activated in accordance with the increased expression of HO-1 (Figure 3A,C).

### 3.3. JNK and PI3K/Akt Regulated HO-1 Expression in RAW 264.7 Cells

In order to identify signaling molecules including MAPKs and PI3K/Akt might be related with the TOWE- and TOEE-induced HO-1 upregulation, RAW 264.7 cells were treated with various concentrations of agents for 4 h and phosphorylated status of each signaling molecule was measured by Western blot analysis.

**Figure 4:**
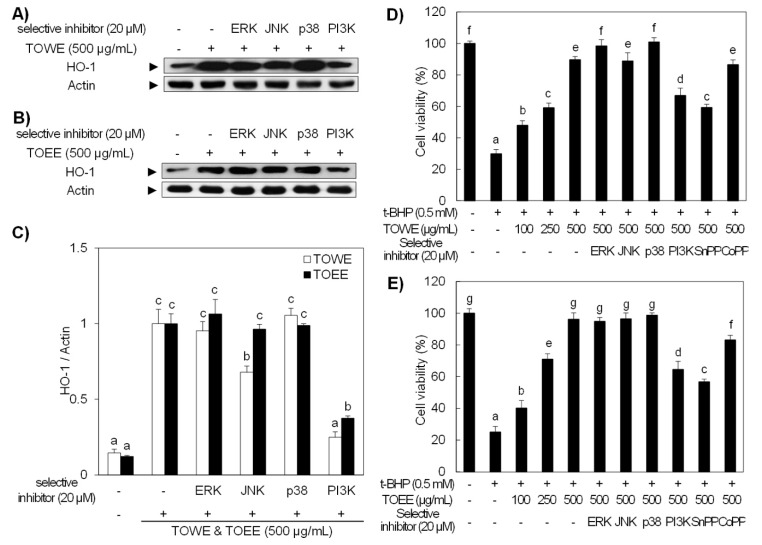
TOWE and TOEE induced HO-1 expression was abolished by the treatment of selective inhibitors, which was confirmed by t-BHP-induced oxidative damage in RAW 264.7 cells. (**A**,**B**) RAW 264.7 cells were treated with 20 μM of selective inhibitors for PI3K/Akt and MAPK signaling molecules in the presence of 500 μg/mL of TOWE and TOEE. The selective inhibitors for JNK and PI3K attenuated TOWE-induced HO-1 expression, while TOEE-induced HO-1 expression was only abolished by a PI3K selective inhibitor. The HO-1 protein expression was analyzed by Western blot analysis. (**C**) The relative induction of HO-1 was quantified by densitometry, and actin was used as an internal control. (**D**,**E**) The antioxidative potential of TOWE and TOEE scavenged the t-BHP-induced oxidative damage in RAW 264.7 cells. RAW 264.7 cells were treated with various concentrations of TOWE or TOEE for 12 h in the presence or absence of each selective inhibitor or inducer. The cells except untreated were exposed to 0.5 mM t-BHP for 3 h. The data represent the mean ± standard deviation of triplicate experiments. The values sharing the same superscript are not significantly different at *p* < 0.05 by Duncan’s multiple range test. t-BHP, tert-butyl hydroperoxide; SnPP, tin protoporphyrin; CoPP, cobalt protoporphyrin; PI3K, phosphoinositide 3-kinase.

As shown in Figure 3B, Akt was activated by both extracts treatment and JNK was only phosphorylated by TOWE treatment, while ERK and p38 were not given any visible effect. These results indicate that the PI3K/Akt and JNK signaling pathways play an important role in the HO-1 expression in RAW 264.7 cells. In addition, selective inhibitors for each signaling molecule were applied in order to prove these results. Figure 4A showed a selective inhibitor for PI3K downregulated both extracts-induced HO-1 expression, while JNK inhibitor only attenuated TOWE-initiated HO-1 expression, respectively. It has been reported that the induction of HO-1 expression is regulated by many different signaling molecules including MAPKs and PI3K/Akt [29]. Khayandirobilide A from *Khaya senegalensis* ameliorated inflammatory responses through the inhibition of inflammatory mediators and the induction of p38 MAPK/Nrf2-mediated HO-1 expression in RAW 264.7 and BV2 cells [30]. *Melaleuca alternifolia* concentrate (MAC) showed an anti-inflammatory effect through HO-1 induction through p38 and JNK pathways in RAW 264.7 cells [30]. The antioxidative potential was strengthened by HO-1 upregulation by luteolin and luteolin-7-*O*-glucoside through the modulated p38 and JNK activation in RAW 264.7 cells [31]. These results suggest that TOWE and TOEE induced HO-1 expression is regulated by the PI3K and JNK signaling molecules in RAW 264.7 cells.

### 3.4. Pretreatment of TOWE and TOEE Protected RAW 264.7 Cells against Oxidative Stress-Induced Cell Death

Endogenously generated ROS in response to external stimuli have been known for their implications with many physiological disorders such as tumorigenesis, cardiovascular diseases, neurodegenerative disorders and inflammatory diseases [1]. Overproduced ROS can cause severe damage to cellular protein, lipid, carbohydrates, and DNA. Loss of cell function or cell death can be led by accumulated damage induced by intracellular ROS. In this study, a stable organic hydroperoxide, t-BHP, was employed in order to induce excessive ROS production, which mitochondrial dysfunction and apoptosis could be happening as a result of oxidative stress. A previous study suggested that t-BHP exposure to RAW 264.7 cells sharply abolished cell viability with excessive intracellular ROS generation, which was counteracted with the treatment of luteolin and luteolin-7-*O*-glucoside [32]. RAW 264.7 cells were pretreated with 500 μg/mL of TOWE and TOEE for 12 h to induce HO-1 expression with selective inhibitors for MAPKs or PI3K signaling molecule. In addition, SnPP and CoPP, selective inhibitor or inducer of HO-1, were also employed to identify the role of HO-1 against oxidative stress-initiated cytotoxic damage. As shown in Figure 4D,E, sharply increased cytotoxicity was significantly attenuated by TOWE and TOEE treatment in a dose-dependent manner. The cytoprotective potential was not exerted on JNK (only by TOWE treatment), PI3K and SnPP treated cells, which means diminished HO-1 expression. Consequently, these results suggest that HO-1 induction by TOWE and TOEE, mediated by Nrf2 activation, alleviated the t-BHP initiated oxidative damage through the regulation of JNK and PI3K signaling pathways in RAW 264.7 cells.

## 4. Conclusions

*T. officinale* has been consumed in folk medicine to treat various ailments, such as heartburn, dyspepsia, and liver disorders. This study tried to evaluate the antioxidative potential and its mechanism of both TOWE and TOEE, in the RAW 264.7 cell line. TOWE and TOEE potently induced one kind of metabolizing enzyme, HO-1, which was in accordance with the nuclear accumulation of transcription factor, Nrf2. Moreover, TOWE induced HO-1 expression through the activation of PI3K/Akt and JNK, while TOEE induced HO-1 expression by PI3K/Akt activation. The antioxidative potential of induced HO-1 was estimated in t-BHP treated RAW 264.7 cells and the cell viability was significantly recovered by both extracts treatment. Consequently, HO-1 expression by TOWE and TOEE treatment attenuated oxidative damage through the regulation of Nrf2/MAPK/PI3K signaling cascades in RAW 264.7 cells.
